# Clinical response and pharmacokinetics of bendamustine as a component of salvage R-B(O)AD therapy for the treatment of primary central nervous system lymphoma (PCNSL)

**DOI:** 10.1186/s12885-018-4632-y

**Published:** 2018-07-09

**Authors:** Therasa Kim, He Yun Choi, Hyun-Seo Lee, Sung-Hoon Jung, Jae-Sook Ahn, Hyeoung-Joon Kim, Je-Jung Lee, Hee-Doo Yoo, Deok-Hwan Yang

**Affiliations:** 1 College of Pharmacy and Research Institute of Pharmaceutical Sciences, Seoul National University, 1 Gwanakro, Gwanakgu, Seoul, 08826 Republic of Korea; 2 Research Center for Cancer Immunotherapy, Chonnam National University Hwasun Hospital, 322 Seoyangro, Hwasun, Jeollanamdo, 58128 Republic of Korea; 3 Department of Hematology-Oncology, Chonnam National University Hwasun Hospital, 322 Seoyangro, Hwasun, Jeollanamdo, 58128 Republic of Korea; 4 Department of Biostatistics and Bioinformatics, Pharma Partnering Inc., 74 Olympicro, Songpagu, Seoul, 05556 Republic of Korea

**Keywords:** Bendamustine, CSF, Pharmacokinetics, Primary CNS lymphoma, Salvage therapy

## Abstract

**Background:**

A relatively high proportion of patients diagnosed with primary CNS lymphoma will experience recurrent disease, yet therapy options are limited in salvage therapy. This is the first study to evaluate a bendamustine-based combination regimen for the treatment of relapsed/refractory PCNSL and to characterize bendamustine pharmacokinetics in the human CSF.

**Methods:**

Patients received bendamustine 75 mg/m^2^ for two days as part of R-B(O)AD administered intravenously every 4 weeks for up to 4 cycles. Response and adverse events of the regimen were assessed. A sparse sampling strategy and population based modeling approach was utilized for evaluation of plasma and CSF levels of bendamustine.

**Results:**

Ten patients were enrolled into study of whom 70% were of refractory disease and with high IELSG prognostic risk scores. The ORR of R-BOAD was 50% (95% CI, 0.24 to 0.76) with one patient achieving CR and four PR. Primary toxicity of the regimen was reversible myelosuppression, mostly grade 3 or 4 neutropenia. The C_max_ mean for plasma and CSF were 2669 ng/mL and 0.397 ng/mL, respectively, and patients with response at deep tumor sites displayed higher trends in peak exposure. Pharmacokinetic data was best described by a four-compartment model with first-order elimination of drug from central plasma and CSF compartments.

**Conclusions:**

R-BOAD is an effective salvage option for PCNSL, but with significant hematologic toxicity. Bendamustine CSF levels are minimal; however correspond to plasma exposure and response.

**Trial registration:**

ClinicalTrials.gov NCT03392714; retrospectively registered January 8, 2018.

**Electronic supplementary material:**

The online version of this article (10.1186/s12885-018-4632-y) contains supplementary material, which is available to authorized users.

## Background

Primary central nervous system lymphoma (PCNSL) is a rare form of CNS malignancy representing 2–4% of all primary CNS tumors and is of mainly diffuse large B-cell lymphoma (DLBCL) origin [1, 2]. Despite high sensitivity to radiation and chemotherapy, one third of the patient population is refractory to first-line therapy and up to half of the responders will relapse after remission from initial treatment, mostly within the CNS [3, 4]. Salvage therapy will be required for a significant percentage of the PCNSL population, yet there is no current standard of therapy in the relapsed/refractory (R/R) setting due to the lack of data from randomized clinical trials. High dose methotrexate (HD-MTX) re-challenge in patients with previously MTX responsive disease is a feasible option reporting high response rates, [5, 6] and numerous single or combination therapy regimens have been examined in small prospective studies with overall response rates ranging from 30 to 55% [7–9].

Bendamustine is a bifunctional alkylating agent possessing both alkylator and antimetabolite properties from a mechlorethamine moiety and benzimidazole ring, respectively. [10] Recently, several case reports have suggested that bendamustine has modest clinical activity as single agent therapy against relapsed PCNSL with reasonable tolerability [11, 12]. However, the effect of this agent as part of combination salvage therapy in patients with PCNSL has not been established. The purine analog-like properties of bendamustine are thought to augment the apoptotic effects of pyrimidine analogs such as cytarabine, and synergy against DLBCL cell lines has been shown to be greatest when administered sequentially [13] where therapeutic impact has been validated in several trials involving mantle cell lymphoma patients [14, 15]. Similarly, vincristine has documented efficacy against lymphoid malignancies when used in combination with bendamustine in both in vitro and clinical studies [16, 17].

Although the role of rituximab in PCNSL has some controversy, a recent meta-analysis has shown that additional use of the CD20-targeted monoclonal antibody in initial therapy correlates with higher response rates [18], and other studies in the salvage setting have reported positive outcomes, albeit a more modest response in prospectively conducted trials [19, 20]. Based on the demonstrated activity and proposed additive mechanisms of these chemotherapeutic agents, we investigated the bendamustine-based combination regimen R-B(O)AD in patients with refractory or relapsed primary CNS lymphoma. Evidence from previous preclinical tissue distribution studies and single agent intravenous drug therapy trials in CNS malignancies suggests that bendamustine penetrates brain and tumor tissue [12, 21–23], and while cerebrospinal fluid (CSF) drug concentrations are commonly used as a surrogate marker of CNS delivery, there are no clinical data available on the pharmacokinetics (PK) of bendamustine in the CSF. In light of rarity of the disease and difficulties in obtaining extensive data samples, a nonlinear mixed-effects modeling approach was considered appropriate for drug evaluation. Thus, we evaluated the PK of plasma and CSF drug levels through a population based model approach in a R/R PCNSL cohort with the goals to define the currently unknown PK profile of bendamustine in the CSF and to further characterize the relationship between plasma and CSF drug levels, and the influence of exposure on response to therapy.

## Methods

### Study eligibility

Eligible patients were ≥ 19 years of age with PCNSL of DLBCL origin diagnosed by CNS lesion tissue biopsy, and in relapse or refractory to frontline chemotherapy or radiation, with confirmed evidence of disease progression by contrast enhanced magnetic resonance imaging (MRI). Additional requirements were Eastern Cooperative Oncology Group (ECOG) performance status 0–2 and adequate hematologic and organ function including absolute neutrophil count (ANC) ≥ 1000/uL, platelets ≥100,000/uL, total bilirubin ≤1.5 x upper limit of normal (ULN), transaminases ≤3 x ULN, and serum creatinine ≤2.0 x ULN. Patients with uncontrolled infection, therapy with myelosuppressive chemotherapy or biologic therapy < 21 days prior to registration, persistent toxicities ≥ grade 3 from prior chemotherapy, history of thromboembolic episodes ≤3 months prior to registration, active hepatitis B or C with uncontrolled disease, or with active other malignancy requiring treatment that would interfere with assessments of lymphoma response to protocol treatment were excluded from enrollment.

### Study design and treatment

This was a prospective, open-label, pilot study investigating the safety and efficacy of the bendamustine-based combination regimen R-B(O)AD, designed to define CSF and plasma PK profiles of bendamustine in R/R patients. All patients received either R-BOAD or R-BAD intravenously (rituximab 375 mg/m^2^ on day 1; vincristine 1.4 mg/m^2^ on day 1, omitted in patients ≥70 years of age due to risk of neurotoxicity; bendamustine 75 mg/m^2^ over 1 h on days 2 and 3; cytarabine 1000 mg/m^2^ over 3 h on days 2–4; dexamethasone 20 mg on days 1–4), every 4 weeks up to 4 cycles. Initial reduction of bendamustine or cytarabine dosage was allowed if deemed necessary by the physician due to elderly age or poor performance status. Subcutaneous granulocyte colony-stimulating factor support was administered post chemotherapy on starting day 7 of the cycle in all patients until ANC ≥ 500/uL. Treatment cycles were delayed until hematologic parameters allowed for the next cycle of therapy (i.e. ANC ≥1000/uL, platelets ≥75,000/uL). If a cycle was postponed > 1 week due to hematologic toxicity bendamustine and cytarabine doses were reduced by was 25% in subsequent cycles. For vincristine, drug dose was reduced by 50% in the case of moderate neurotoxicity (grade 2), and if severe (grade 3 or 4) was discontinued for all subsequent cycles.

### Efficacy and safety measurements

Baseline assessments included physical and neurological examination, laboratory studies, ocular slit lamp and CSF examination, and contrast enhanced cranial MRI. Once enrolled into study, response was assessed after every two cycles of therapy or at any time point where progression was suspected. Evaluation of response to treatment was based on criteria defined by Abrey et al. [24]. Patients who did not respond to the first two cycles of therapy were discontinued from study. The efficacy of the combination regimen was determined by ORR, defined as patients with complete (CR) or partial response (PR). Two-sided 95% confidence intervals (CIs) are given for efficacy endpoint ORR, using the Wilson method for small sample size [25]. After completion of study treatment, patients with PR or CR were reassessed every three months. Safety was assessed after each treatment cycle by documentation of adverse events based on the NCI Common Terminology Criteria (version 4.0) through physical examination and clinical laboratory results.

### Pharmacokinetic assessments

Utilizing sparse sampling strategies, plasma and CSF samples for PK analyses were acquired in pairs on the first day of bendamustine administration (day 2 of combination regimen), one pair per cycle per patient, with a cumulative target collection of three observations per time point. Sampling time points (0 min, 30 min, 1 h, 3 h, and 8 h post completion of bendamustine infusion) were selected based on previously published plasma pharmacokinetic studies showing near complete elimination of drug within 8 h of infusion completion [26, 27]. Additional time points were investigated if deemed necessary to clarify plasma and CSF exposure profiles of bendamustine, but each patient was not to exceed three sampling time points during the entire course of therapy. At designated time points 5 mL of whole blood and 2 mL of CSF by lumbar puncture were drawn into evacuated EDTA and clear tubes, respectively, and immediately placed on ice. Within 1 h, blood samples were centrifuged at 1500 rpm for 10 min at 4 °C, supernatant withdrawn and transferred into 100 uL aliquots. Aliquots of plasma and CSF samples were stored at − 70 °C until quantification. Bendamustine drug concentrations were determined by validated liquid chromatography/tandem mass spectrometry (LC-MS/MS) methodology with modifications, and a lower limit of quantification (LLOQ) of 0.05 ng/mL for CSF and 5 ng/mL for plasma [28]. The inter-day coefficients of variation for the assay of bendamustine concentrations were ≤ 3.0% and ≤ 9.4% for plasma and CSF, respectively.

### CSF exposure estimates and PK model

CSF exposure was estimated as C_max,CSF_/C_max,plasma_ and AUC_CSF_/AUC_plasma_ ratios. Non-compartmental methods were used to calculate the area under the concentration–time curve (AUC_0-inf_) in WinNonlin, version 5.2 (Pharsight, St. Louis, MO, USA). The relationship between drug levels and tumor location, and interim responder status (i.e. response after two cycles of treatment or first suspected progression) was assessed by classifying patients according to observed maximum plasma and CSF concentrations, and the involvement of deep structures.

Population PK analyses were performed with NONMEM software, version 7.3 (ICON Development Solutions, Ellicott City, MD, USA) using the first-order conditional estimation method (FOCE) and ADVAN6 routine. Processing of NONMEM output and generation of plots were conducted using Xpose 4.5.3 and Sigma plot 12.0 (SYSTAT, Salano, California, USA). Plasma concentration of bendamustine was best described by a two-compartment model, parameterized for central (V_1_) and peripheral (V_2_) compartment volumes of distribution with inter-compartmental (Q_1_) and elimination clearance (CL). For addition of the CSF compartment, a biophase reservoir was applied between the central plasma and CSF compartment. The structural model for CSF concentration data was parameterized for biophase (V_3_) and CSF (V_4_) compartment volumes of distribution with CSF elimination clearance (CL_csf_) and inter-compartmental clearances Q_2_ and Q_3_. Inter-individual variability (IIV) was modeled with an exponential error model and residual variability (RV) was assessed using a proportional error model.

The most appropriate pharmacostatistical model was selected on the basis of goodness-of-fit plots, precision of estimates, and the likelihood ratio test using NONMEM generated objective function values (OFV). Goodness-of-fit plots included observed and predicted individual profiles, population predicted estimates, and conditional weighted residuals [29]. Precision of the population estimates was evaluated on the basis of relative standard errors (RSE, %) and inter-individual variability was estimated in terms of the coefficient of variance (CV, %). The accuracy and robustness of the final population model was evaluated using a non-parametric bootstrap analysis. Replication sets of the original data were generated (*N* = 1000) to which the final population model was re-fit and stability of the model was evaluated by comparing final model parameter estimates to the median and 90% CIs of the bootstrap replicates.

## Results

### Patient characteristics and treatment

Between January 2016 and March 2017, ten patients were enrolled into study at a single center in South Korea. All subjects had CNS lymphoma of DLBC origin and a majority of the patients were with poor prognostic scores based on the International Extranodal Lymphoma Study Group (IELSG) risk scoring system [30]. All patients had previously received high dose methotrexate as part of initial treatment and most patients were of refractory disease. Of the three relapsed patients, one patient entered study at second relapse. Twenty-seven cycles of R-B(O)AD were administered, at a median of three cycles per patient, and vincristine was omitted in four patients. Four patients were treated with initial dosage reductions in bendamustine and cytarabine by 25%, due to elderly age (> 70 years; one patient 78 years of age received cytarabine 500 mg/m^2^). Chemotherapy was delayed in five of the 27 cycles but further dose reduction was not required as no subsequent cycle was postponed longer than 7 days. G-CFS was given to all patients. Patient characteristics and responses are summarized in Table 1.

**Table 1:** Patient characteristics (*N* = 10) and responses

Patient ID No.	Sex/ Age (years)	ECOG PS	IELSG score*	Disease state	Previous therapy	Tumor location	R-B(O)AD cycles completed	Final response	PFS/OS (months)
1	F/68	2	5	Ref	HDMTX+ AraC	D; periventricular, basal ganglia	3	PD	1.8/7.3
2	F/55	2	4	Ref	HDMTX+ AraC	D; periventricular, corpus callosum	2	PD	2.5/6.8
3	M/75	1	4	Rel	HDMTX + WBRT; MPV-A	ND; L optic nerve	4	CR	21.7/> 21.7
4	M/42	2	3	Ref	HDMTX	D; basal ganglia	2	PD	1.6/9.1
5	M/78	2	3	Rel	HDMTX+ AraC	ND; L parietal	4	PR	6.9/> 6.9
6	F/55	2	4	Ref	HDMTX+ AraC	ND; L frontal, R temporal	3	PD	2.8/3.3
7	F/47	1	2	Ref	HDMTX+ AraC	D^**t**^; L frontal, periventricular	1	PR	2.8/2.8
8	F/73	2	5	Rel	HDMTX+ AraC	ND; L frontal	4	PR	4.4/4.4
9	M/75	2	4	Ref	HDMTX+ AraC	ND^**t**^; L frontal	2	SD	4.2/> 4.2
10	M/65	2	5	Ref	HDMTX	D^**t**^; L frontal, basal ganglia	2	PR	3.9/3.9

Abbreviations: M, male; F, female; PS, performance status; Rel, relapsed; Ref, refractory; HDMTX, high dose methotrexate; AraC, cytarabine; WBRT, whole brain radiotherapy; MPV-A, methotrexate, vincristine, procarbazine, cytarabine; D, deep; ND, non-deep; L, left; R, right; CR, complete response; PR, partial response; SD, stable disease; PD, progressive disease; PFS, progression free survival; OS, overall survival

*IELSG risk = intermediate (IELSG score 2–3), high (IELSG score 4–5)

^t^Patients with leptomeningeal involvement in recurrent disease

### Efficacy and safety

**Fig. 1:**
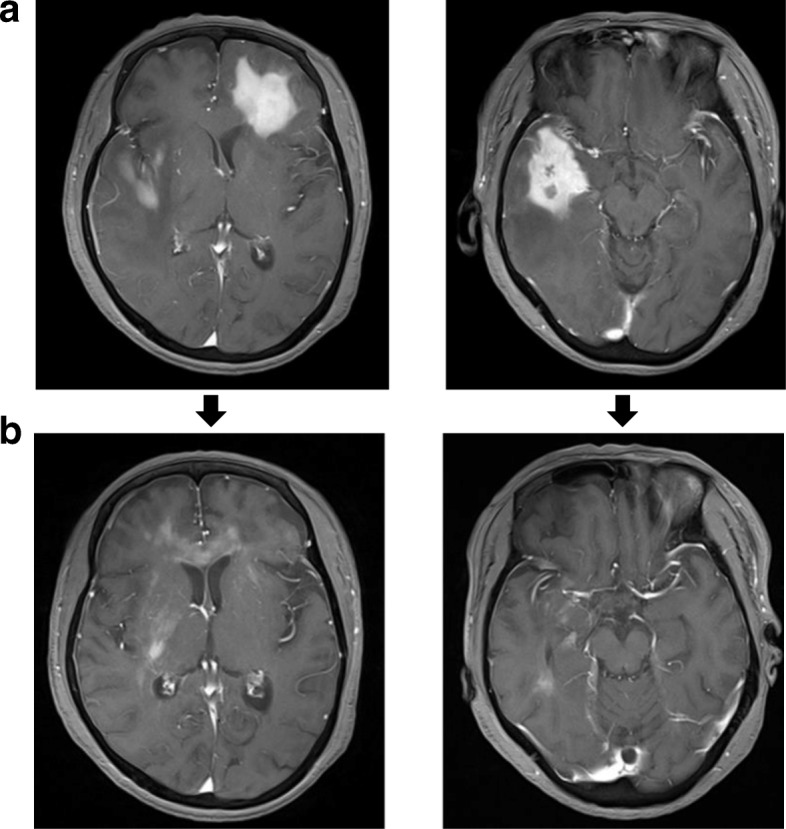
Resolution of multi-focal (left frontal and right temporal lobe) disease in a 65 year old patient (ID No. 6) **a**) before and **b**) after two cycles of R-BOAD at interim analysis

The ORR of R-B(O)AD was 50% (95% CI, 0.24 to 0.76), one patient achieving CR (10%) and four PR (40%). The CR patient showed lymphomatous infiltration in the left optic nerve with thickening visible on MRI that completely resolved after the second cycle of R-BAD. All subjects who progressed on salvage therapy were patients of refractory disease. One of these patients showed near complete resolution of multiple tumor sites during interim analysis (Fig. 1), however developed new lesions after the third cycle of therapy. Four of the five patients who progressed during study treatment received WBRT post salvage therapy. Primary toxicity of the combination regimen was reversible myelosuppression, mostly grade 3 or 4 neutropenia (89% of cycles). Grade 3 febrile neutropenia was observed in 33%, grade 3 or 4 thrombocytopenia in 85%, and grade 1 or 2 anemia in 70% of treatment cycles. The most common non-hematological toxicities were nausea and diarrhea, 30 and 19%, respectively, mostly grade 1 or 2. Infection (mostly pneumonia) was observed in three patients, all requiring antibiotic therapy, and one resulted in treatment related mortality. This was a 73 year old patient who received four cycles of R-BAD therapy with documented partial reduction in tumor after cycle two, and during cycle four developed urinary catheter related *Klebsiella pneumoniae* infection and progressive bronchopneumonia on chest imaging, with delayed hematologic recovery.

### Pharmacokinetic exposure data

**Fig. 2:**
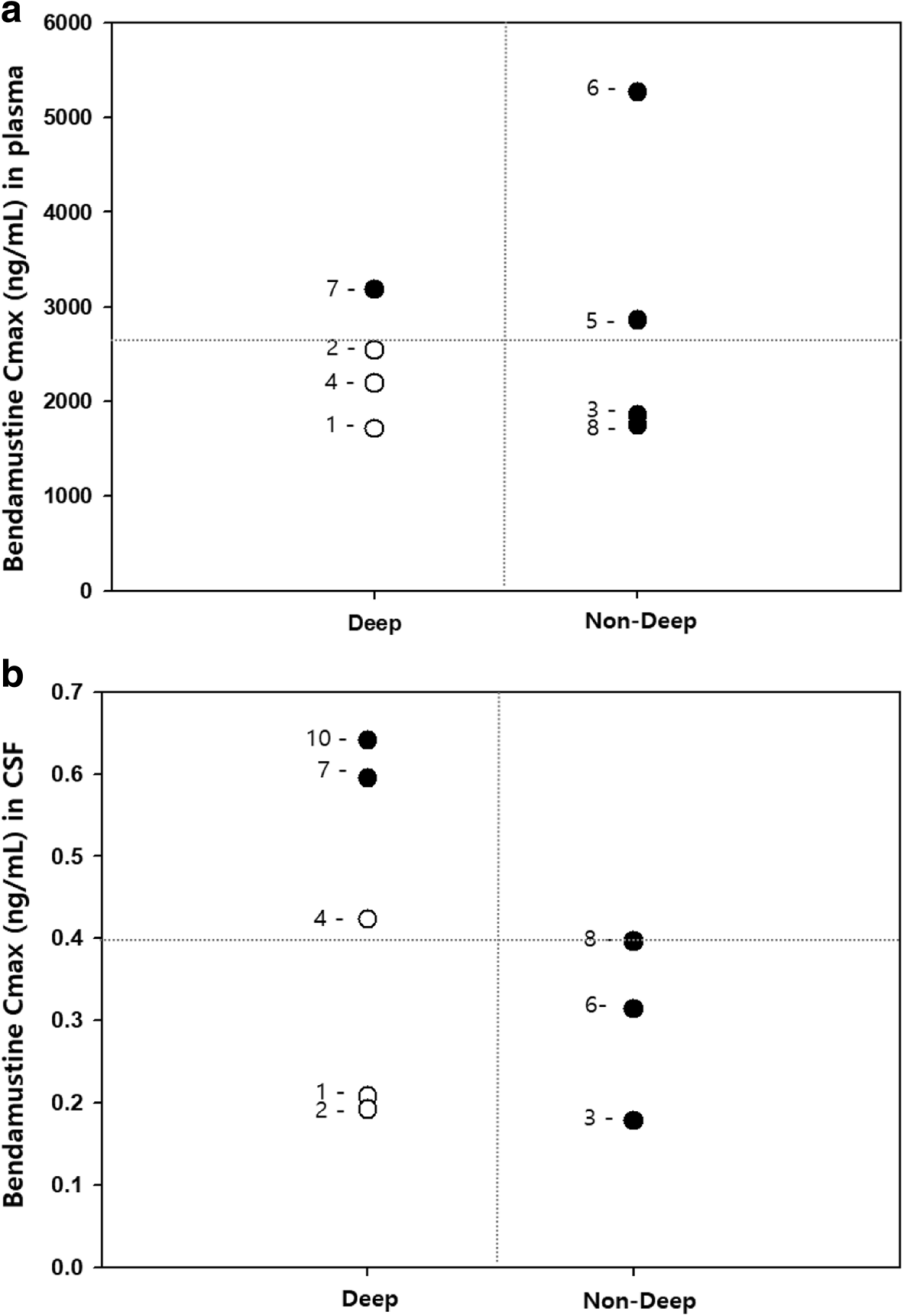
Relationship between interim response status and **a**) plasma C_max_ and **b**) CSF C_max_ bendamustine concentrations, and tumor location. Deep structures include periventricular regions, basal ganglia, brainstem, and cerebellum regions. Closed circles (•) represent responders and open circles (°) non-responders. Patient identification numbers are notated and the dashed line depicts mean C_max_ values for plasma and CSF. Bendamustine exposure was not significantly higher for the patient resulting in treatement related death (ID No. 8)

A total of 28 plasma and 16 CSF samples were collected. Time of maximum concentration (t_max_) was found at the end of infusion (t_max,plasma_ = 1 h) for plasma and at 0.5 h after end of infusion (t_max,csf_ = 1.5 h) for CSF. The C_max_ mean for plasma was 2669 ng/mL (SD ±1176 ng/mL) and for CSF 0.397 ng/mL (SD ±0.160 ng/mL). CSF/plasma exposure ratios were calculated to be 0.015 and 0.025% for C_max_ and AUC_0-inf_, respectively. Individual observations at C_max,plasma_ and C_max,csf_ were available for eight patients. All patients who showed an interim response were subjects with involvement of non-deep structures as classified by Ferreri et al. [30] and those displaying tumor regression at deep sites possessed higher trends in C_max,plasma_ and C_max,csf_ values (Fig. 2).

### Population pharmacokinetic model

**Fig. 3:**
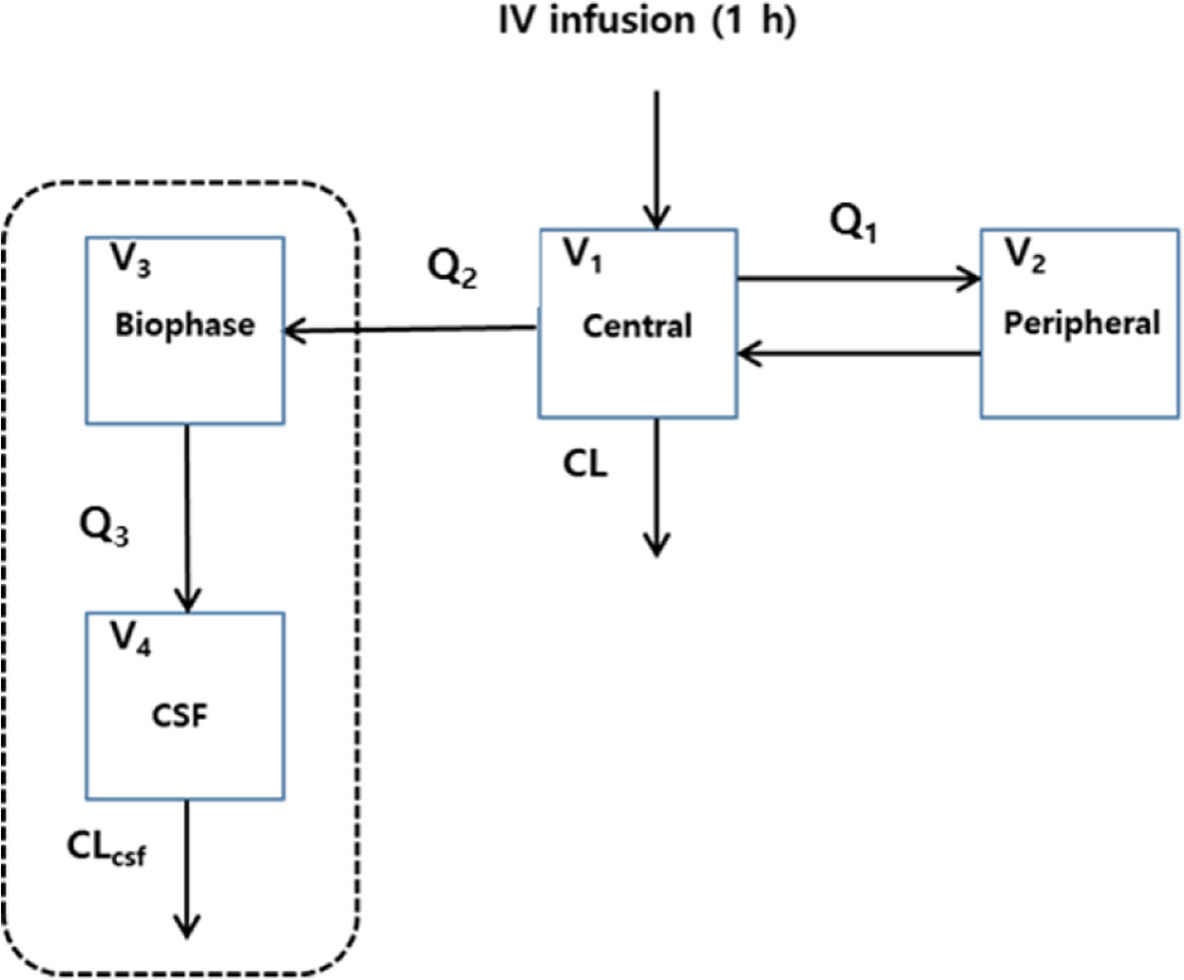
Schematics of structural model used for bendamustine. Abbreviations: V, volume of distribution; Q, inter-compartmental clearance; CL, clearance of central plasma compartment; CL_csf_, clearance of CSF compartment

**Fig. 4:**
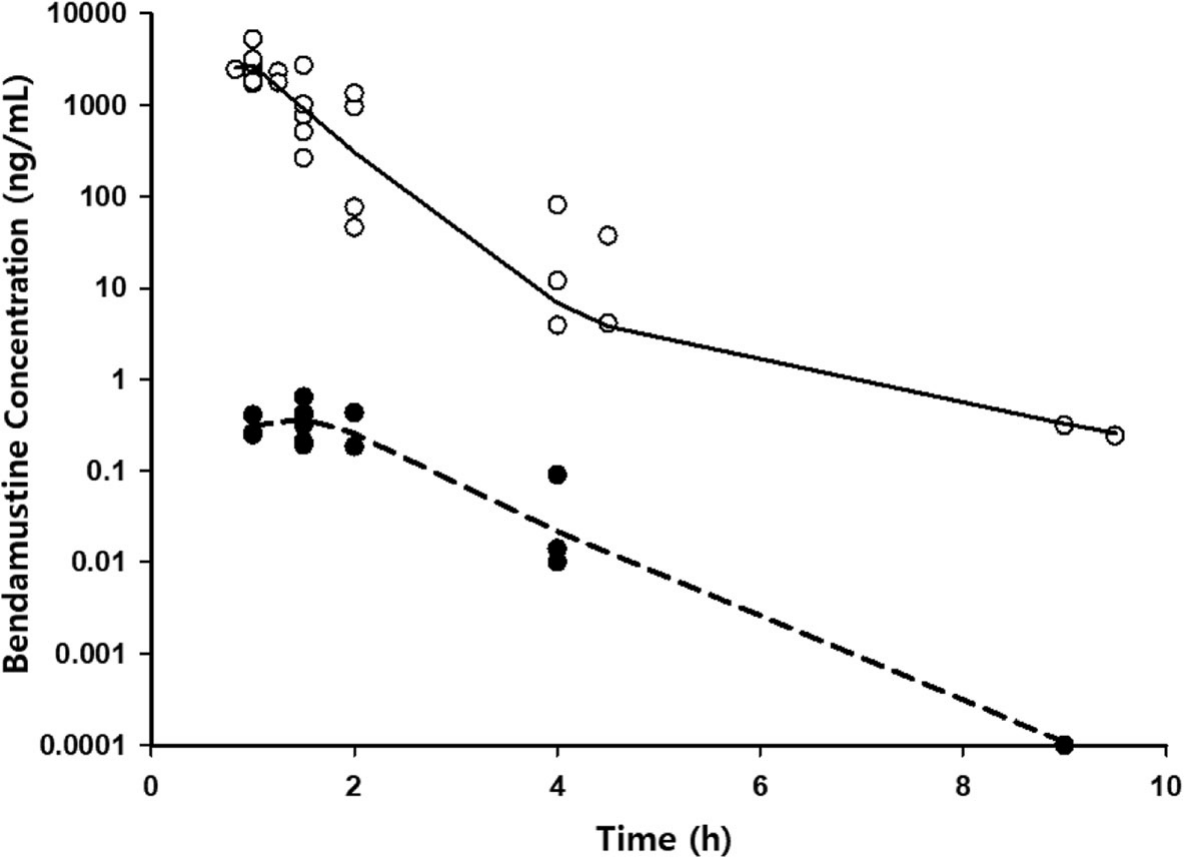
Bendamustine concentration-time profiles. Circles represent observed values for plasma (•) and CSF (°) drug levels. Best-fit curves from the final population PK model are shown for plasma () and CSF (−---)

Pharmacokinetic data was best fit by a four-compartment model incorporating two plasma compartments (central and peripheral) with drug distributing from central plasma into an intermediate biophase reservoir and then into a final CSF compartment, with first-order elimination of drug from both central plasma and CSF compartments (Fig. 3). The biophase compartment was required to account for the delay in time to reach maximum drug concentrations in the CSF after infusion completion, compared to the immediate peak in plasma achieved at the end of IV infusion. The overall volume of distribution in plasma (V_plasma_ = V_1_ + V_2_ = 19.7 L) was similar to previously reported values (~ 20 L) as was elimination clearance from the central plasma compartment (32.5 L/hr. for patient with BSA = 1.675) [26, 27]. Inter-individual variability in pharmacokinetic variables was moderate, with coefficient of variance values near 40%. A proportional model was employed to assess residual variability for which CV% was 17%. Overall, observed bendamustine concentrations in plasma and CSF were adequately fit by population predicted median values, indicating ability of the final model to describe central tendencies (Fig. 4). Estimates for the final model were similar to bootstrap replicates and were contained within the 90% CI, representing absence of significant bias. The PK parameter mean estimates with associated standard errors and the 90% bootstrap confidence intervals are presented in Table 2.

**Table 2:** Population PK model parameter estimates and nonparametric bootstrap 90% confidence intervals

Parameter	Estimate	RSE (%) /CV (%)	Bootstrap Replicates		
			Median	CI (90%)	
V_1_	14.9	19.2	14.1	4.9	20.7
CL	32.5	10.5	31.4	23.9	39.2
V_2_	0.508	14.4	0.455	0.186	0.846
Q_1_	0.238	15.3	0.205	0.041	0.660
V_3_	0.323	9.8	0.322	0.186	0.442
Q_2_	0.569	15.8	0.569	0.344	0.836
V_4_	0.032	40.9	0.032	0.014	0.041
Q_3_	0.793	16.8	0.795	0.573	1.360
CL_csf_	0.075	43.5	0.075	0.059	0.140
IIV V_1_	0.230	42.9	0.220	0.011	0.480
IIV CL	0.089	39.9	0.086	0.014	0.190
RV	0.420	17.4	0.390	0.210	0.620

Abbreviations: RSE (%, for structural parameter estimates), relative standard error; CV (%, for IIV), coefficient of variance; CI, confidence interval; V (L), volume of distribution; CL (L/h), elimination clearance; Q (L/h), inter-compartmental clearance; CLcsf (L/h), CSF compartment clearance; IIV, inter-individual variability; RV, residual variability

## Discussion

This was a prospective pilot trial investigating a bendamustine-based combination regimen in patients with R/R primary CNS lymphoma. Recurrent disease is difficult to treat in that progression is usually rapid and aggressive leading to significant impairment in performance status and neurological deterioration, a limited number of salvage strategies exist, and survival outcomes are suboptimal despite additional therapy. The 50% ORR of the study regimen falls within the range of efficacy prospectively observed with current salvage regimens utilized in R/R PCNSL. A previous case series report demonstrated a best response rate of 50% to single agent bendamustine therapy with acceptable toxicity but was retrospective in nature and showed a relatively short lived response. In this study, utilization of mechanistically augmenting chemotherapeutic agents resulted in an active salvage regimen with remarkable effects observed on imaging in patients showing response. Such activity may largely be attributed to the anticipated synergy effect of combination bendamustine and cytarabine, considering the majority of the patients had progressed despite previous treatment with cytarabine as a part of induction therapy.

However, such synergistic effects of the combination also lead to significant marrow suppression, and hematologic toxicity observed with R-B(O)AD was considerable with grade 3 or 4 neutropenia and thrombocytopenia experienced in > 85% of treatment cycles. The rate of toxicity observed was somewhat higher than that reported in a previous study investigating treatment of mantle-cell non-Hodgkin lymphoma patients with bendamustine (70 mg/m^2^) and cytarabine (800 mg/m^2^) combination therapy [14], and may be explained by ethnic and disease differences in the study population. Severe infection was observed in three patients, all with involvement of the lungs, one patient with underlying COPD disease and another with a history of fungal pneumonia. Due to significant myelosuppression despite the use of prophylactic growth factor support, study protocol was later amended with reduction in cytarabine dosage to 500 mg/m^2^ and stricter criteria for dose adjustments in the case of severe cytopenias.

To our knowledge, this is the first study to characterize the pharmacokinetics of bendamustine in human CSF. Given multiple sampling of the CSF through lumbar puncture is not feasible for both medical and ethical reasons, a sparse sampling method and population PK approach was employed with collection of CSF at different time points among patients. CSF concentrations of bendamustine were minimal compared to plasma values with an AUC exposure ratio of 0.025%. Although absolute values of CSF drug levels were much lower than those in plasma, higher trends in maximum peak concentrations of drug in the CSF showed correlation to tumor response, particularly in patients with lymphoma involvement of deep brain structures. Among patients with observations sampled at t_max,plasma_ and t_max,csf_, all subjects with tumors outside deep regions showed significant tumor regression regardless of PK concentrations. This is in line with the IELSG scoring system in which disease involvement of deep structures is an independent prognostic variable associated with poor survival [30].

The inclusion of a biophase reservoir between the central plasma and CSF compartment allowed for the observed delay in time to peak concentrations of the CSF, and may be representative of anatomical structures that are part of the CSF macrocirculation but are farther from the sampled lumbar puncture site. The concentration time profile of the biophase reservoir was simulated using the population PK model and drug exposure was predicted to be similar to that of the CSF compartment (Additional file 1: Figure S1). Clearance of bendamustine from the CSF compartment was rapid with an elimination half-life t_1/2, csf_ = 0.30 h calculated from model parameter estimates. Bendamustine like other nitrogen mustards has limited stability and undergoes degradation by hydrolysis, which increases in the presence of water and higher temperatures [31, 32]. Such chemical properties of bendamustine may contribute to the extensive elimination of drug observed in the CSF.

A previous tissue distribution study of IV ^14^C-bendamustine demonstrated radioactivity in brain tissue of mice, rats, and dogs, suggesting permeability of drug through the blood brain barrier (BBB) [33]. Despite the association observed between bendamustine concentrations in the CSF and drug activity, it is doubtful that such low concentrations found in our study are representative of true drug levels in the brain parenchyma. This may partially be explained by differences in drug penetration through the BBB and the blood-CSF barrier due to variations in the endothelium and transporter expression, in which case CSF concentrations would not serve as an adequate marker of drug delivery to the tumor location. Such discrepancies between drug levels in the CSF and brain tissue have been reported for several chemotherapeutic agents. Temsirolimus, an mTOR inhibitor, has demonstrated drug levels in tissue above those in the plasma of glioma patients, yet in another study negligible CSF drug levels were observed in CNS lymphoma patients [34, 35]. Single agent rituximab therapy has shown clinical activity in disease of the CNS but also possesses poor detectable drug levels in the CSF after IV administration [20]. Therapeutic levels of such drugs in the brain parenchyma are thought to be achieved by penetration of a BBB that is with compromised integrity due to the highly disordered and permeable vasculature of the infiltrating tumor [36]. In these settings drug concentrations are expected to decrease with increasing distance from the tumor bulk.

## Conclusion

A relatively high proportion of patients with PCNSL will experience progression of disease, yet the number of prospective trials on salvage therapy remains small due to the rarity of disease and rapidly progressive nature. It is accepted that salvage therapy is beneficial and significantly improves survival in comparison to palliative care. Although this study reports data from a limited number of patients, it supports the use of a bendamustine-based combination regimen as an option for salvage therapy, especially in patients who are no longer chemo-sensitive to methotrexate or those who have developed cumulative renal or neurotoxicity from treatment. Hematologic toxicity of the regimen is significant but manageable with dose reduction and supportive care. A lower dosage of cytarabine at 500 mg/m^2^ may be more feasible to avoid prolongation of significant marrow suppression and will be investigated in a Phase II study. Evaluation of plasma and CSF data with development of a population PK model shows CSF drug levels are low with rapid decline and are unlikely to be an accurate predictor of drug concentrations at the tumor site, thus should not be utilized as a surrogate of CNS drug delivery. However, trends in higher peak bendamustine concentrations in both plasma and CSF were observed for patients who showed response to treatment in deep tumor locations.

## Additional file

Additional file 1: **Figure S1.** Simulations of bendamustine concentration-time profiles for compartments included in final PK model. C1, central plasma compartment; C2, peripheral plasma compartment; C3, biophase compartment; C4, CSF compartment. (DOCX 53 kb)

## Abbreviations

AraC: Cytarabine
CI: Confidence interval
CL: Clearance of central plasma compartment
CLcsf: Clearance of CSF compartment
CR: Complete response
CSF: Cerebrospinal fluid
CV: Coefficient of variance
D: Deep
DLBCL: Diffuse large B-cell lymphoma
F: Female
HDMTX: High dose methotrexate
IELSG: International Extranodal Lymphoma Study Group
IIV: Inter-individual variability
L: Left
M: Male
MPV-A: Methotrexate, vincristine, procarbazine, cytarabine
ND: Non-deep
ORR: Overall response rate
OS: Overall survival
PCNSL: Primary central nervous system lymphoma
PD: Progressive disease
PFS: Progression free survival
PK: Pharmacokinetics
PR: Partial response
PS: Performance status
Q: Inter-compartmental clearance
R: Right
R/R: Relapsed/refractory
Ref: Refractory
Rel: Relapsed
RSE: Relative standard error
RV: Residual variability
SD: Stable disease
V: Volume of distribution
WBRT: Whole brain radiotherapy
