# Supplementary figures and images for: Clinical response and pharmacokinetics of bendamustine as a component of salvage R-B(O)AD therapy for the treatment of primary central nervous system lymphoma (PCNSL)
Source: BMC Cancer. 2018 Jul 9;18:729. doi: 10.1186/s12885-018-4632-y (PMC6038347; doi:10.1186/s12885-018-4632-y)

**Supplementary Figure S1.**


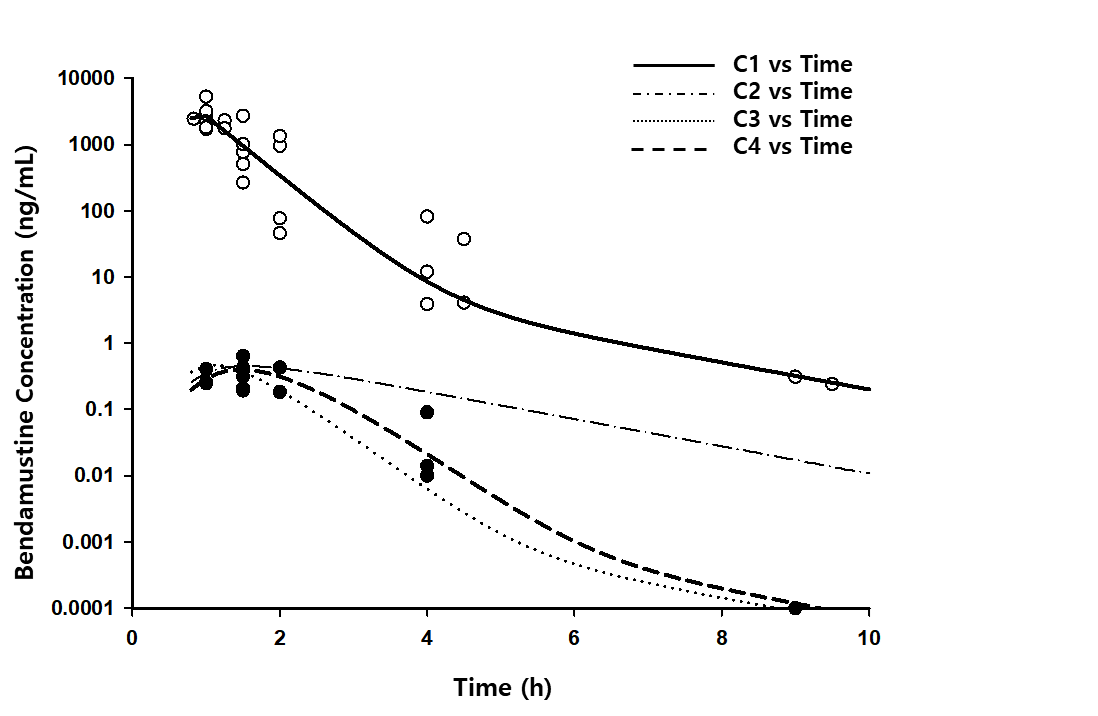

Supplement: Supplementary file 1 — Figure S1. Simulations of bendamustine concentration-time profiles for compartments included in final PK model. C1, central plasma compartment; C2, peripheral plasma compartment; C3, biophase compartment; C4, CSF compartment. (DOCX 53 kb) [file 12885_2018_4632_MOESM1_ESM.docx]
